# Systematics of Atomic Orbital Hybridization of Coordination Polyhedra: Role of f Orbitals

**DOI:** 10.3390/molecules25143113

**Published:** 2020-07-08

**Authors:** R. Bruce King

**Affiliations:** Department of Chemistry, University of Georgia, Athens, GA 30602, USA; rbking@uga.edu

**Keywords:** coordination polyhedra, hybridization, atomic orbitals, f-block elements

## Abstract

The combination of atomic orbitals to form hybrid orbitals of special symmetries can be related to the individual orbital polynomials. Using this approach, 8-orbital cubic hybridization can be shown to be sp^3^d^3^f requiring an f orbital, and 12-orbital hexagonal prismatic hybridization can be shown to be sp^3^d^5^f^2^g requiring a g orbital. The twists to convert a cube to a square antiprism and a hexagonal prism to a hexagonal antiprism eliminate the need for the highest nodality orbitals in the resulting hybrids. A trigonal twist of an *O_h_* octahedron into a *D*_3*h*_ trigonal prism can involve a gradual change of the pair of d orbitals in the corresponding sp^3^d^2^ hybrids. A similar trigonal twist of an *O_h_* cuboctahedron into a *D*_3*h*_ anticuboctahedron can likewise involve a gradual change in the three f orbitals in the corresponding sp^3^d^5^f^3^ hybrids.

## 1. Introduction

In a series of papers in the 1990s, the author focused on the most favorable coordination polyhedra for sp^3^d*^n^* hybrids, such as those found in transition metal complexes. Such studies included an investigation of distortions from ideal symmetries in relatively symmetrical systems with molecular orbital degeneracies [1] In the ensuing quarter century, interest in actinide chemistry has generated an increasing interest in the involvement of f orbitals in coordination chemistry [2,3,4,5,6,7]. This has prompted me to revisit such issues, adding the new feature of f orbital involvement as might be expected to occur in structures of actinide complexes and relating hybridization schemes to the polynomials of the participating orbitals.

The single s and three p atomic orbitals have simple shapes. Thus, an s orbital, with the quantum number l = 0, is simply a sphere equivalent in all directions (isotropic), whereas the three p orbitals, with quantum number l = 1, are oriented along the three axes with a node in the perpendicular plane. For higher nodality atomic orbitals with l > 1 nodes the situation is more complicated since there is more than one way of choosing an orthogonal 2 l + 1 set of orbitals. In this connection, a convenient way of depicting the shapes of atomic orbitals with two or more nodes is by the use of an orbital graph [8]. Such an orbital graph has vertices corresponding to its lobes of the atomic orbital and the edges to nodes between adjacent lobes of opposite sign. Orbital graphs are necessarily bipartite graphs in which each vertex is labeled with the sign of the corresponding lobe and only vertices of opposite sign can be connected by an edge. For clarity in the orbital graphs depicted in this paper, only the positive vertices are labeled with plus (+) signs. The unlabeled vertices of the orbital graphs are considered negative.

**Figure 1:**
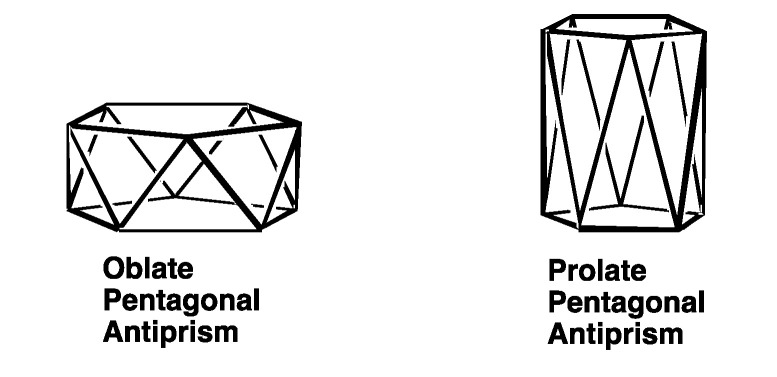
The oblate and prolate pentagonal antiprisms on which the two sets of five equivalent d orbitals are based indicating the amounts of compression and elongation, respectively.

Table 1 shows the orbital graphs for the commonly used set of five d orbitals as well as the corresponding orbital polynomials. The orbital graphs for four of this set of five d orbitals are squares, whereas that for the fifth d orbital is a linear configuration with three vertices and two edges. The exponent of the variable z corresponds to l-m, i.e., 2-m for this set of d orbitals. This set of d orbitals is not the only possible set of d orbitals. Two alternative sets of d orbitals have all five d orbitals of equivalent shape corresponding to rectangular orbital graphs oriented to exhibit five-fold symmetry [9,10,11,12] One set of these equivalent d orbitals is based on an oblate (compressed) pentagonal antiprism, whereas the other set of equivalent d orbitals is based on a prolate (elongated) pentagonal prism (Figure 1). These sets are useful for structures with five-fold symmetry corresponding to symmetry point groups such as *D*_5*d*_, *D*_5*h*_, and *I_h_* in the structures studied here. For the icosahedral group *I_h_* all five d orbitals belong to the five-dimensional H_g_ irreducible representation.

Two different sets of f orbitals are used depending on the symmetry of the system (Table 2) [13]. The general set of f orbitals is used except for systems with high enough symmetry to have three-dimensional irreducible representations. This general set consists of a unique f{*z*^3^} orbital with a linear orbital graph and *m* = 0, an f{*xz*^2^,*yz*^2^} pair with a double square orbital graph and *m* = ± 1, an f{*xyz*, *z*(*x*^2^ − *y*^2^)} pair with a cube orbital graph and *m* = ±2, and an f{*x*(*x*^2^ − 3*y*^2^),*y*(3*x*^2^ − *y*^2^)} set with a hexagon orbital graph and *m* = ±3. For systems having point groups with three-dimensional irreducible representations, such as the octahedral *O_h_* and the icosahedral *I_h_* point groups, the so-called cubic set of f orbitals is used. The cubic set of f orbitals consists of the triply degenerate f{*x*^3^,*y*^3^,*z*^3^} set with linear orbital graphs and a quadruply degenerate f{*xyz*, *x*(*z*^2^ − *y*^2^), *y*(*z*^2^ − *x*^2^), *z*(*x*^2^ − *y*^2^)} set.

**Figure 2:**
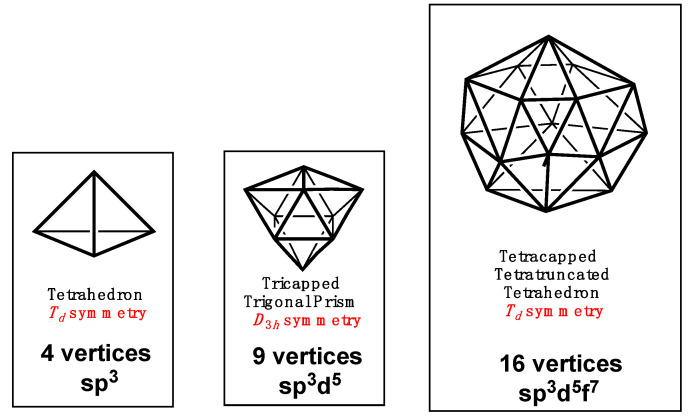
The most spherical deltahedra corresponding to filled 4-orbital sp^3^, 9-orbital sp^3^d^5^, and 16-orbital sp^3^d^5^f^7^ manifolds.

Fully using the complete sp^3^, sp^3^d^5^, and sp^3^d^5^f^7^ manifolds in hybridization schemes should lead to most nearly spherical polyhedra (Figure 2). For the four-orbital sp^3^ system such a polyhedron is, of course, the familiar regular tetrahedron. For the 16-orbital sp^3^d^5^f^7^ manifold the most spherical polyhedron is the tetracapped tetratruncated tetrahedron, also of *T_d_* point group symmetry. Such a 16-vertex deltahedron is the largest Frank-Kasper polyhedron where a Frank-Kasper polyhedron is a deltahedron with only degree 5 and degree 6 vertices with no pair of adjacent degree 6 vertices [14]. The tetracapped tetratruncated tetrahedron is rarely found in experimental molecular structures but has been recently realized in the central Rh_4_B_12_ polyhedron in the rhodaborane Cp*_3_Rh_3_B_12_H_12_Rh(B_4_H_9_RhCp*), synthesized by Ghosh and co-workers [15]. There is no similarly symmetrical most spherical 9-vertex polyhedron. The most spherical 9-vertex deltahedron is the *D*_3*h*_ tricapped trigonal prism found experimentally in the MH_9_^2−^ (N = Tc, Re) anions [16,17].

## 2. Polyhedral Hybridizations

### 2.1. Polyhedra with Tetragonal Symmetry

The simplest configuration with tetragonal symmetry is the square, found as a coordination polyhedron in diamagnetic four-coordinate complexes of d^8^ metals such as Ni(II), Pd(II), Pt(II), Rh(I), and Ir(I). Since a square is a two-dimensional structure in an *xy* plane, the polynomials of the atomic orbitals for square hybridization cannot contain a *z* variable. This limits the p orbital involvement in square hybridization to the p{*x*} and p{*y*} orbitals so that a d orbital is required for square hybridization. This d orbital can be either the d{*x*^2^ − *y*^2^} or d{*xy*} orbital, depending on whether the *x* and *y* axes are chosen to go through the vertices or edge midpoints, respectively, of the square (Table 3).

**Figure 3:**
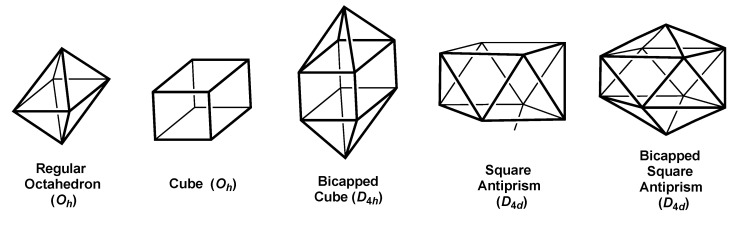
Tetragonal polyhedra with at least one *C*_4_ rotation axis.

The smallest three-dimensional polyhedron with tetragonal symmetry of interest in this context is the regular octahedron with the *O_h_* point group containing both three-fold and four-fold axes (Figure 3). This is the most frequently encountered polyhedron in coordination chemistry and is also the most spherical 6-vertex *closo* deltahedron in the structures of boranes and related species. The *O_h_* point group contains two- and three-dimensional irreducible representations. Under octahedral symmetry, the five d orbitals split into a triply degenerate T_2g_{*xz*,*yz*,*xy*} set and a doubly degenerate E_g_{*x*^2^ − *y*^2^,*xy*} set. Octahedral hybridization supplements the four-orbital sp^3^ set with the doubly degenerate E_g_{*x*^2^ − *y*^2^,*xy*} set of d orbitals.

An octahedral coordination complex can be stretched or compressed along a four-fold axis thereby reducing the symmetry from *O_h_* to *D*_4*h*_ by removing the three-fold axes. This corresponds to the Jahn–Teller effect [18] leading to a structure that can be designated as a square bipyramid, analogous to pentagonal and hexagonal bipyramids discussed later in this paper. This reduction in symmetry of an octahedral metal complex splits the triply degenerate T_1u_ p orbitals into a doubly degenerate E_1u_{*x*,*y*} set and a non-degenerate A_2u_{*z*} orbital and the doubly degenerate E_g_{*x*^2^ − *y*^2^,*xy*} set of orbitals into non-degenerate A_1g_{*z*^2^} and B_1g_{*x*^2^ − *y*^2^} orbitals.

The most symmetrical polyhedron for an 8-coordinate complex is the cube, which is the dual of the regular octahedron and, thus, also exhibits the *O_h_* point group (Figure 3). Considering the cube as two squares stacked on top of each other at a distance leading to the three-fold symmetry elements of the *O_h_* point group leads to a simple way of deriving the atomic orbitals for cubic hybridization. Thus, to form a set of cubic hybrid orbitals, the atomic orbitals for square hybridization are supplemented by the four atomic orbitals in which the polynomials for square hybridization are multiplied by *z*. In this way, a set of pd^2^f orbitals with each atomic orbital having one more node than the corresponding orbital in the sp^2^d orbital set for square hybridization is added to the sp^2^d square hybridization to form an (sp^2^d)(pd^2^f) = sp^3^d^3^f set for cubic hybridization. This shows that one f orbital, namely the A_2u_{*xyz*} orbital with a cube orbital graph (Table 2), is required for cube hybridization. This suggests that 8-coordinate ML_8_ complexes with cubic coordination are likely to be restricted to lanthanide and actinide chemistry, particularly the latter. Reducing the symmetry of the cube from *O_h_* to *D*_4*h*_ by elongation or compression along a four-fold axis thereby eliminating the three-fold symmetry does not eliminate the need for an f orbital in the hybridization of an ML_8_ complex.

The need for an f orbital in the hybridization for an 8-coordinate metal complex can be eliminated by converting a cube into a square antiprism by rotating a square face of a cube 45° around the C_4_ axis thereby changing the *O_h_* point group into *D*_4*d*_ (Figure 3 and Table 3). The sp^3^d^4^ hybridization for the square antiprism omits the d{*z*^2^} orbital.

Capping the square faces of a square antiprism retaining the *D*_4*d*_ symmetry leads to the most spherical *closo* deltahedron for 10-vertex borane derivatives, such as B_10_H_10_^2−^ (Figure 3). The d{*z*^2^} and f{*z*^3^} orbitals, both of which lie along the four-fold *z* axis, are added to th sp^3^d^4^ hybridization of the square antiprism to give the sp^3^d^5^f{*z*^3^} hybridization of the bicapped square antiprism. In a similar way the d{*z*^2^} and f{*z*^3^} orbitals can be added to the sp^3^d^3^f hybridization of the cube to give the sp^3^d^4^f^2^ hybridization of the bicapped cube with D_4*d*_ symmetry.

### 2.2. Polyhedra with Pentagonal Symmetry

The simplest structure with pentagonal symmetry is the planar pentagon. Using a set of five orbitals with only *x* and *y* in the orbital polynomial leads to sp^2^{*x*,*y*}d^2^(*x*^2^ − *y*^2^,*xy*} hybridization for planar pentagon coordination with minimum l values. Either the prolate or oblate sets of equivalent d orbitals (Figure 1) can provide alternative d^5^ hybridization for a planar pentagon structure, but with higher combined l values for the five-orbital set and poor overlap between the ligand orbitals and those of the central atom.

**Figure 4:**
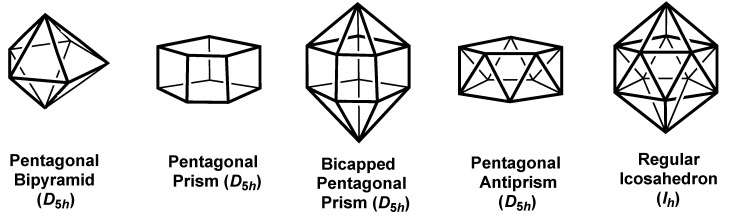
Pentagonal polyhedra with at least one *C*_5_ rotation axis.

The smallest three-dimensional polyhedron of interest with pentagonal symmetry is the *D_5h_* pentagonal bipyramid (Figure 4). The sp^3^d^3^ scheme for pentagonal bipyramidal coordination supplements the five-orbital sp^2^{*x*,*y*}d^2^(*x*^2^ − *y*^2^,*xy*} hybridization for the equatorial pentagon with the p{*z*} and d{*z*^2^} orbitals for the linear sub-coordination of the axial ligands perpendicular to the equatorial planar pentagon (Table 4).

The 10-vertex pentagonal prism of *D*_5*h*_ symmetry (Figure 4) is encountered experimentally in the endohedral trianions M@Ge_10_^3–^ (M = Fe [19], Co [20]), isolated as K(2,2,2-crypt)^+^ salts and structurally characterized by X-ray crystallography. Considering the pentagonal prism as two pentagons stacked on top of each other to preserve the *D*_5*h*_ symmetry supplements the sp^2^{*x*,*y*}d^2^(*x*^2^ − *y*^2^,*xy*} of the planar pentagon with an additional set of five orbitals with the orbital polynomials multiplied by *z*, i. e. the p{*z*}d^3^*(xz,yz*}f^2^{*xyz*,*z*(*x*^2^ − *y*^2^)} set. Thus, the set of 10 orbitals for the pentagonal prism are sp^3^d^4^{*x*^2^ − *y*^2^,*xy*,x*z,*y*z*} f{*xyz*,*z*(*x*^2^ − *y*^2^)} without involvement of the d{z^2^} orbital. Capping the pentagonal prism in a way to preserve the *D*_5*h*_ symmetry adds the d{*z*^2^} and f{*z*^3^} orbitals to the hybrid leading to an sp^3^d^5^f^3^{*z*^3^,*xyz*,*z*(*x*^2^ − *y*^2^)} set for the resulting 12-vertex bicapped pentagonal prism.

Twisting one pentagonal face of a pentagonal prism 36° around the *C*_5_ axis leads to the pentagonal antiprism of *D*_5*d*_ symmetry (Figure 4). Unlike the analogous conversion of the cube to the square antiprism, this process does not change the sp^3^d^4^f^2^ hybridization. Capping the two pentagonal faces of the pentagonal antiprism while preserving five-fold symmetry leads to the bicapped pentagonal antiprism. This adds the d(*z*^2^) and f(*z*^3^) orbitals to give an sp^3^d^5^f^3^ 12-orbital hybridization scheme. This process is exactly analogous to the conversion of the square antiprism to the bicapped square antiprism discussed above.

The special *D_nd_* symmetry of an *n*-gonal antiprism is preserved by compression or elongation along the major *C_n_* axis to give oblate or prolate polyhedra, respective, as illustrated in Figure 1 for the pentagonal antiprisms representing the five equivalent d orbitals. The regular icosahedron, so important in several areas of chemistry, including polyhedral borane chemistry, is a special case of the bicapped pentagonal antiprism with a specific degree of compression/elongation to add three-fold symmetry to the *D_5d_* symmetry of the bicapped pentagonal antiprism to give the full icosahedral point group *I_h_*. This ascent in symmetry combines some of the irreducible representations of the *D*_5*h*_ point group to give irreducible representations of higher degeneracy. As a result, the A_1g_ + E_1g_ + E_2g_ irreducible representations coalesce into the single five-fold degenerate H_g_ representation of the icosahedral point group. The five d orbitals belong to this H_g_ representation in icosahedral symmetry. Similarly, the three f orbitals of the sp^3^d^5^f^3^ icosahedral hybridization belong to the single T_2u_{*x*^3^,*y*^3^,z^3^} irreducible representation of the *I_h_* point group considering the cubic set of f orbitals (Table 2).

### 2.3. Polyhedra with Hexagonal Symmetry

The simplest structure with hexagonal symmetry is the planar hexagon itself. The restriction of the atomic orbitals to those containing no *z* term in their polynomials requires the use of a single f atomic orbital to supplement the planar five-orbital sp^2^{*x*,*y*}d^2^(*x*^2^ − *y*^2^,*xy*} set with either f orbital with a planar hexagon orbital graph, namely the f{*x*(*x*^2^ − 3*y*^2^)} or the f{*y*(3*x*^2^ − *y*^2^)} orbital depending on the {*x*,*y*,*z*} coordinate system chosen.

**Figure 5:**
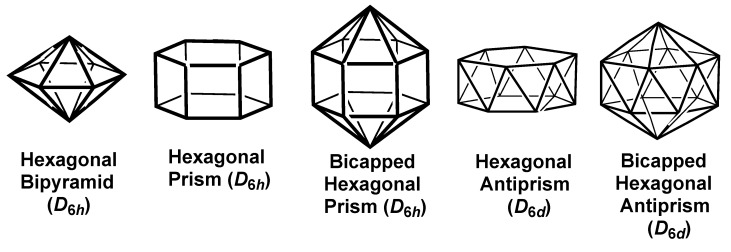
Hexagonal polyhedra with at least one *C*_6_ rotation axis.

The smallest three-dimensional figure with hexagonal symmetry is the 8-vertex hexagonal bipyramid (Figure 5). The sp^3^d^3^f set of hybrid orbitals for the hexagonal bipyramid is obtained by supplementing the sp^2^{*x*,*y*}d^2^{*x*^2^ − *y*^2^,*xy*}f{*x*(*x*^2^ − 3*y*^2^)} set of orbitals for the equatorial planar hexagon with the p{*z*} and d{*z*^2^} pair for the two additional axial vertices. The hexagonal bipyramid shares with the cube (see above) the feature of not arising from any sp^3^d^4^ hybridization scheme but instead requiring an f orbital in a sp^3^d^3^f hybridization scheme.

The atomic orbitals required to form a prism combine those for the polygonal face having only *x* and *y* in their orbital polynomials with an equal number of orbitals in which the polynomials obtained by multiplying the orbital polynomials for the planar face by the *z* variable. Thus, the sp^2^{*x*,*y*}d^2^{*x*^2^ − *y*^2^,*xy*}f{*x*(*x*^2^ − 3*y*^2^)} orbitals corresponding to the hexagonal face of the hexagonal prism is supplemented by the p{*z*^3^}d^2^{*xz*,*yz*}f^2^{*xyz*,*z*(*x*^2^ − *y*^2^)}g{*xz*(*x*^2^ − 3*y*^2^)} set of six orbitals. This leads to the interesting observation that a single g orbital corresponding to the B_2g_ irreducible representation is required to provide a set of 12 hybrid orbitals oriented towards the vertices of a *D*_6*h*_ hexagonal prism (Table 5). Not surprisingly, this g orbital has a hexagonal prism orbital graph. Capping the hexagonal prism in a way to preserve the *D*_6*h*_ symmetry adds the d{*z*^2^} and f{*z*^3^} orbitals to the hybrid leading to an sp^3^d^5^f^3^{*z*^3^,*xyz*,*z*(*x*^2^ − *y*^2^)} for the resulting 12-vertex bicapped pentagonal prism.

Twisting one hexagonal face of a hexagonal prism 30° around the *C*_6_ axis leads to the hexagonal antiprism of *D*_6*d*_ symmetry (Figure 5). Going from the *D*_6*h*_ symmetry of the hexagonal prism to the *D*_6*d*_ symmetry of the hexagonal antiprism in a 12-vertex hexagonal symmetry system eliminates the need for a g orbital in the set of 12 atomic orbitals forming the corresponding hybrid orbitals. This is analogous to the conversion of the cube requiring sp^3^d^3^f hybridization to the square antiprism with sp^3^d^4^ hybridization not requiring an f orbital by rotation one square face 45° relative to its opposite partner. Capping the hexagonal faces of the hexagonal antiprism in a way to preserve its six-fold symmetry leads to the bicapped hexagonal antiprism, which is of significance as being the most spherical *closo* 14-vertex deltahedron in borane chemistry. This capping process adds d{*z*^2^} and f{*z*^3^} orbitals to the 12-orbital hybridization scheme for the hexagonal antiprism leading to a sl^3^d^5^f^5^ hybridization scheme for the bicapped hexagonal antiprism not requiring g orbitals (Table 5).

### 2.4. Trigonal Twist Processes in Polyhedral of Octahedral Symmetry

**Figure 6:**
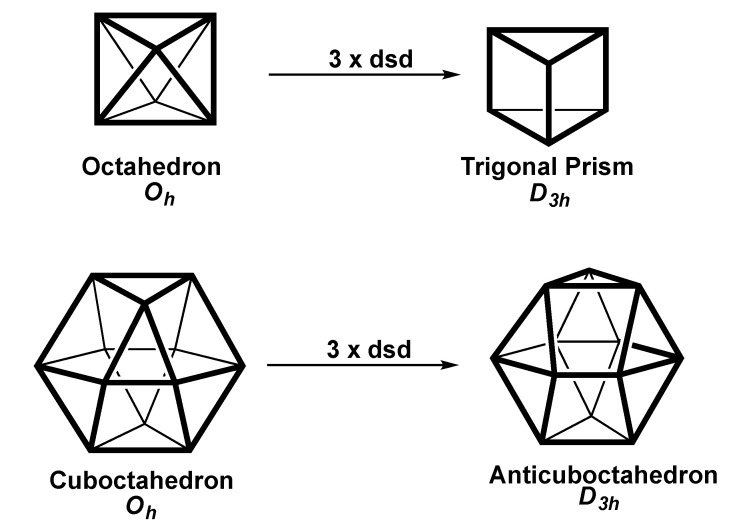
Polyhedra with octahedral (*O_h_*) symmetry and their conversions by a triple diamond-square-diamond process to a polyhedron with *D*_3*h*_ symmetry.

The octahedron and the cuboctahedron are the simplest two polyhedra with triangular faces and *O_h_* point group symmetry having both three-fold and four-fold rotation symmetry axes. The process of converting prisms of *n*-fold symmetry (*n* = 4, 5, and 6 including the cube as a special tetragonal prism) to the corresponding antiprisms involves a twist of (180/*n*)° of an *n*-fold face around the unique *n*-fold axis. Analogous processes can be applied to the three-fold axes in the regular octahedron and icosahedron. Such a twist process converts the *O_h_* regular octahedron into the *D*_3h_ trigonal prism and the *O_h_* cuboctahedron into the *D*_3*h*_ anticuboctahedron (Figure 6). These processes can be regarded as trigonal twists involving three symmetry related diamond-square-diamond transformations. For octahedral coordination complexes of the type M(bidentate)_3_ involving bidentate chelating ligands variations of this process are designated as Bailar twists [21] or Ray-Dutt [22] twists.

Such trigonal twist processes can be studied by first relaxing the symmetry of the original *O_h_* polyhedron to *D*_3*d*_, which is the subgroup of *O_h_* obtained by removing the *C*_4_ axes. Under this subgroup, the octahedron can be viewed as a trigonal antiprism with the *z* axis corresponding to the *C*_3_ axis rather than the *C*_4_ axis in the discussion above of polyhedral with *C*_4_ symmetry. Under *D_3d_* symmetry rather than the higher *O_h_* symmetry and with the different location of the *z* axis, the degenerate E_g_{*xz*,*yz*} pair of d orbitals as well as the E_g_(*x*^2^ − y^2^,*xy*}pair can be used for octahedral sp^3^d^2^ hybridization (Table 6). When the twist reaches the stage of the *D*_3*h*_ trigonal prism only the E´{*xz*,*yz*} pair of atomic orbitals is available for the sp^3^d^2^ hybridization scheme. Thus, the trigonal twist of an octahedron to a trigonal prism involves replacement of the E_g_{*x*^2^ − y^2^,*xy*}pair of orbitals in the *D_3d_* octahedral sp^3^d^2^ hybrids with the E´{*xz*,*yz*} pair of orbitals in the *D*_3*h*_ trigonal prismatic hybrids.

The sp^3^d^5^f^3^ hybridization for a cuboctahedron using its full *O_h_* symmetry taking the *z* axis as a *C*_4_ axis supplements the full 9-orbital sp^3^d^5^ set with the triply degenerate T_2u_{*x*(*z*^2^ − *y*^2^),*y*(*z*^2^ − *x*^2^),*z*(*x*^2^ − *y*^2^)} set of the cubic f orbitals (Table 6). Reducing the symmetry of the cuboctahedron to *D*_3*d*_ and now taking the *C*_3_ axis as the *z* axis splits, the triply degenerate set of f orbitals into a non-degenerate A_1u_{*z*^3^} orbital and a doubly degenerate Eu set of orbitals, which can be either the Eu{*xyz*,z(*x*^2^ − *y*^2^)} or the Eu{*x*(3*x*^2^ − *y*^2^),*y*(3*x*^2^ − *y*^2^)} set. Thus, for the cuboctahedron, like the octahedron under *D_3d_* symmetry, there are two alternative pairs of the required highest l value orbitals in the minimum l value hybrid that can be used in the hybrid. Converting the *D_3d_* cuboctahedron to the *D_3h_* anticuboctahedron eliminates the Eu{*x*(3*x*^2^ − *y*^2^),*y*(3*x*^2^ − *y*^2^)} pair of f orbitals from the sp^3^d^5^f^3^ hybrid requiring use of the E´{*xz^2^,yz*^2^} pair.

## 3. Conclusions

The combination of atomic orbitals to form hybrid orbitals of special symmetries can be related to the individual orbital polynomials. Thus, planar hybridizations such as the square, pentagon, and hexagon can only use atomic orbitals with only two variables in their polynomials, conventionally designated as *x* and *y*. Since there are only two orbitals for each non-zero l value and one orbital (the s orbital) for l = 0, square planar hybridization requires one d orbital, pentagonal planar hybridization requires two d orbitals, and hexagonal planar hybridization requires an f orbital as well as two d orbitals. Prismatic configurations with two parallel *n*-gonal faces and *D_nh_* symmetry combine the set of atomic orbitals for the *n*-gonal face with only the two *x* and *y* variables with a set of equal size corresponding to the orbitals having polynomials in which the polynomials of the planar *n*-gon orbitals are multiplied by a third *z* variable. As a result, cubic hybridization can be shown to require an f orbital and hexagonal prismatic hybridization can be shown to require a g orbital. For 8-coordinate systems with four-fold symmetry twisting a square face of a cube with sp^3^d^3^f hybridization by 45° around a *C*_4_ axis to give a square antiprism with sp^3^d^4^ hybridization eliminates the need for an f orbital in the resulting sp^3^d^4^ hybridization. Similarly, for 12-coordinate systems with six-fold symmetry, twisting a hexagonal face of a hexagonal prism with sp^3^d^5^f^2^g hybridization to a hexagonal antiprism eliminates the need for a g orbital in the resulting sp^3^d^5^f^3^ hybridization. A trigonal twist of an *O_h_* octahedron into a *D*_3*h*_ trigonal prism can involve a gradual change of the pair of d orbitals in the sp^3^d^2^ hybrids. A similar trigonal twist of an *O_h_* cuboctahedron into a *D*_3*h*_ cuboctahedron can likewise involve a gradual change in the three f orbitals in the sp^3^d^5^f^3^ hybridization.

## Figures and Tables

**Table 1:** The polynomials, angular functions, and orbital graphs for the five d orbitals.

Polynomial	Angular Function	Appearance and Orbital Graph	Shape
*xy* *x*^2^ − *y*2 *xz* *yz*	sin^2^ sin sin^2^ cos^2^ sin cos cos sin cos sin	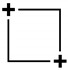	square
2*z*^2^ − *r*^2^ (abbreviated as *z*^2^)	(3cos^2^ − 1)	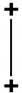	linear

**Table 2:** The polynomials, angular functions, and orbital graphs for both the general and cubic sets of the seven f orbitals.

|*m*|	Lobes	Shape	Orbital Graph	General Set	Cubic Set
3	6	Hexagon	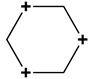	*x*(*x*^2^ − 3*y*^2^) *y*(3*x*^2^ − *y*^2^)	none
2	8	Cube	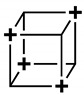	*xyz* *z*(*x*^2^ − *y*^2^)	*xyz* *x*(*z*^2^ − *y*^2^) *y*(*z*^2^ − *x*^2^) *z*(*x*^2^ − *y*^2^)
1	6	Double Square	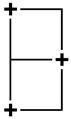	*x*(5*z*^2^ − *r*^2^) *y*(5*z*^2^ − *r*^2^)	none
0	4	Linear	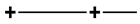	*z*(5*z*^2^ − *r*^2^)	*x* ^3^ *y* ^3^ *z* ^3^

**Table 3:** Hybridization schemes for the tetragonal polyhedra.

Polyhedron	Coord.	Hybridization			
(Symmetry)	No.	Type	s + p	d	f
Square (*D*_4*h*_)	4	sp^2^	A_1g_ + E_u_	B_1g_ {*x*^2^ − *y*^2^}	—
Square Bipy (*D*_4*h*_)	6	sp^3^d^2^	A_1g_ + A_2u_ + E_u_	A_1g_{*z*^2^} + B_1g_ {*x*^2^ − *y*^2^}	—
Octahedron (*O_h_*)	6	sp^3^d^2^	A_1g_ + T_1u_	E_g_{*x*^2^ − *y*^2^,*xy*}	—
Square Prism(*D*_4*h*_)	8	sp^3^d^3^f	A_1g_ + A_2u_ + E_u_	B_1g_ {*x*^2^ − *y*^2^} + E_g_{*xz*,*yz*)	B_2u_{ *z*(*x*^2^ − *y*^2^)}
Cube(*O_h_*)	8	sp^3^d^3^f	A_1g_ + T_1u_	T_2g_{*xy*,*xz*,*yz*}	A_2u_{*xyz*}
Bicapped Cube(*D*_4*h*_)	10	sp^3^d^4^f^2^	A_1g_ + A_2u_ + E_u_	A_1g_{*z*^2^} + B_1g_ {*x*^2^ − *y*^2^} + E_g_{*xz*,*yz*)	A_2u_(*z*^3^} + B_2u_{ *z*(*x*^2^ − *y*^2^)}
Square Antiprism(*D*_4*d*_)	8	sp^3^d^4^	A_1_ + B_2_ + E_1_	E_2_{*x*^2^ − *y*^2^,*xy*} + E_3_{*xz*,*yz*)	—
Bicap Sq Antipr(*D*_4*d*_)	10	sp^3^d^5^f	A_1_ + B_2_ + E_1_	A_1_{*z*^2^} + E_2_{*x*^2^ − *y*^2^,*xy*} + E_3_{*xz*,*yz*)	B_2_{*z*^3^}

**Table 4:** Hybridization schemes for the pentagonal polyhedra.

Polyhedron	Coord.	Hybridization			
(Symmetry)	No.	Type	s + p	d	f
Pentagon (*D*_5*h*_)	5	sp^2^d^2^	A_1_’ + E_1_´	E_2_´{*x*^2^ − *y*^2^}	—
Pent Bipy (*D*_5*h*_)	7	sp^3^d^3^	A_1_´ + A_2_˝ + E_1_´	A_1_´{*z^2^*} *+* E_2_´{*x*^2^ − *y*^2^,*xy*}	—
Pent Prism (*D*_5*h*_)	10	sp^3^d^4^f^2^	A_1_´ + A_2_˝ + E_1_´	E_2_´{*x*^2^ − *y*^2^} + E_1_˝{*xz*,*yz*}	E_2_˝{*xyz*,*z*(*x*^2^ − *y*^2^)}
Bicap Pent Prism(*D*_5*h*_)	12	sp^3^d^5^f^3^	A_1_´ + A_2_˝ + E_1_´	A_1_´{*z^2^*} *+* E_2_´{*x*^2^ − *y*^2^,*xy*} + E_1_˝{*xz*,*yz*}	A_2_˝{*z*^3^} + E_2_˝{*xyz*,*z*(*x*^2^ − *y*^2^)}
Pent Antiprism(*D*_5*d*_)	10	sp^3^d^4^f^2^	A_1g_ + A_2u_ + E_u_	E_1g_{*xz*,*yz*} + E_2g_{*x*^2^ − *y*^2^,*xy*}	E_2u_
Bicap Pent Antipr(*D*_5*d*_)	12	sp^3^d^5^f^3^	A_1g_ + A_2u_ + E_u_	A_1g_{*z*^2^} + E_1g_{*xz*,*yz*} + E_2g_{*x*^2^ − *y*^2^,*xy*}	A_2u_{*z*^3^} + E_2u_
Icosahedron (I_h_)	12	sp^3^d^5^f^3^	A_1g_ + T_1u_	H_g_{all d orbitals}	T_2u_{*x*^3^,*y*^3^,z^3^}

**Table 5:** Hybridization schemes for the hexagonal polyhedra.

Polyhedron	Coord.	Hybridization				
(Symmetry)	No.	Type	s + p	d	f	g
Hexagon (*D_6h_*)	6	sp^2^d^2^f	A_1g_ + E_1u_	E_2g_{*x*^2^ − *y*^2^}	B_1u_{*x*(*x*^2^ − 3*y*^2^)}	—
Hexagonal Bipy(*D_6h_*)	8	sp^3^d^3^f	A_1g_ + A_2u_ + E_1u_	A_1u_{*z*^2^} + E_2g_{*x*^2^ − *y*^2^,*xy*}	B_1u_{*x*(*x*^2^ − 3*y*^2^)}	—
Hexagonal Prism(*D_6h_*)	12	sp^3^d^4^f^3^g	A_1g_ + A_2u_ + E_1u_	E_1g_{*xz*,*yz*} + E_2g_{*x*^2^ − *y*^2^,*xy*}	B_1u_ + E_2u_{*xyz*,*z*(*x*^2^ − *y*^2^)}	B_2g_
Bicap Hex Prism(*D_6_*_h_)	14	sp^3^d^5^f^4^g	A_1g_ + A_2u_ + E_1u_	A_1u_{*z*^2^} + E_1g_{*xz*,*yz*}+E_2g_{*x*^2^ − *y*^2^,*xy*}	A_2u_{*z*^3^} + B_1u_ + E_2u_	B_2g_
Hex Antiprism(*D*_6*d*_)	12	sp^3^d^4^f^4^	A_1_ + B_2_ + E_1_	E_2_{*x*^2^ − *y*^2^,*xy*} + E_5_{*xz,yz*}	E_3_ + E_4_{*xyz*,*z*(*x*^2^ − *y*^2^)}	—
Bicap Hex Antipr(*D*_5*d*_)	14	sp^3^d^5^f^5^	A_1_ + B_2_ + E_1_	A_1_{*z*^2^} + E_2_{*x*^2^ − *y*^2^,*xy*} + E_5_{*xz,yz*}	B_2_(*z*^3^) + E_3_ + E_4_	—

**Table 6:** Hybridization schemes for polyhedra with *O_h_* symmetry and the *D*_3*h*_ polyhedral forme from them by a triple diamond-square-diamond process.

Polyhedron	Coord.	Hybridization			
(Symmetry)	No.	Type	s + p	d	f
Octahedron (*D*_3*d*_)	6	sp^3^d^2^	A_1g_ + A_2u_ + E_u_	E_g_(*x*^2^ − y^2^,*xy*}or E_g_{*xz*,*yz*}	—
Trigonal Prism (*D*_3*h*_)	6	sp^3^d^2^	A_1_´ + A_2_˝ + E_1_´	E˝{*xz*,*yz*}	—
Cuboctahedron (*O_h_*)	12	sp^3^d^5^f^3^	A_1g_ + T_1u_	E_g_(*x*^2^ − y^2^,*xy*} + T_2g_{*xy*,*xz*,*yz*}	T_2u_{*x*(*z*^2^ − *y*^2^),*y*(*z*^2^ − *x*^2^),*z*(*x*^2^ − *y*^2^)}
Cuboctahedron (*D*_3*d*_)	12	sp^3^d^5^f^3^	A_1g_ + A_2u_ + E_u_	A_1g_{*z*^2^} + E_g_(*x*^2^ − y^2^,*xy*} + E_g_{*xz*,*yz*}	A_2u_{*z*^3^} + E_u_
Anticuboctahedron(*D*_3*h*_)	12	sp^3^d^5^f^3^	A_1_´ + A_2_˝ + E_1_´	A_1_´{*z*^2^} + E´(*x*^2^ − y^2^,*xy*} + E˝{*xz*,*yz*}	A_1_´{*x*(*x*^2^ − 3*y*^2^} + E´{*xz*^2^,*yz*^2^}
